# The complete plastome sequence of *Lilium wahingtonianum* Kellogg (Liliaceae)

**DOI:** 10.1080/23802359.2018.1424579

**Published:** 2018-01-11

**Authors:** Hyoung Tae Kim, Ki-Byung Lim

**Affiliations:** a Department of Horticulture, Kyungpook National University, Daegu, Republic of Korea;; b Institute of Agricultural Science and Technology, Kyungpook National University, Daegu, Republic of Korea

**Keywords:** *Lilium washingtonianum*, plastome, phylogeny

## Abstract

*Lilium washingtonianum* Kellogg is native to Western America and grows on slope in high subalpine. In the present study, the complete plastome of *L. washingtonianum* was sequenced. The plastome sequence was 151,967 bp long, with a large single copy region of 81,393 bp, a small single copy region of 17,352 bp, and two inverted repeat regions of 26,611 bp each. Total 133 genes were identified, including 83 coding genes, 8 ribosomal RNAs, 38 transfer RNAs, and 4 pseudogenes. Among four pseudogenes, pseudo *ndhF* gene was the first to report among *Lilium* species so far. The phylogenetic position of *L. washingtonianum* was sister to *L. superbum* but American lilies were not monophyletic.

The genus *Lilium* L. consists of approximately 100 species and the main speciation seems to have occurred after the main landmasses were separated (Pelkonen and Pirttila 2012). In terms of many morphological characteristics, the classification of lilies has been modified for long time (Baker 1871; Wilson 1925; Comber 1949; De Jong 1974;) but is still controversial because of the discordance with phylogenetic analysis using molecular markers (Hayashi and Kawano 2000; Du et al. 2014).

*Lilium washingtonianum* Kellogg is native to Western America and grows on slope in high subalpine (McRae et al. 1998). According to Comber (1949), American lilies fell into section *Pseudolirion* but a clade of American lilies was not strongly supported (Nishikawa et al. 1999; Du et al. 2014) or they were paraphyletic (Hayashi and Kawano 2000; Lee et al. 2011). It seems that these low phylogenetic resolutions were caused by poor informative molecular markers in the phylogenetic studies of lilies and that it is necessary to increase gene numbers to solve the low phylogenetic resolution in genus *Lilium* (Rokas and Carroll 2005).

The complete plastome of *L. washingtonianum* was sequenced in the present study. Total genomic DNA was extracted from fresh leaves of *L. washingtonianum,* which grew from a seed using DNeasy plant mini kit (Qiagen, Valencia, CA, USA) following the protocol of the manufacture and it is stored in Kyungpook University. Total DNA was sequenced using HiSeq 2500 instrument (Illumina, San Diego, CA, USA).

Genes in the plastome were annotated using Geneious (Kearse et al. 2012) by comparing with the previously reported plastome sequences in *Lilium* species. The plastome sequences of 21 *Lilium* and 3 *Fritillaria* species were downloaded from the NCBI database for phylogenetic analysis. A total of 77 genes were extracted from their plastome sequences and each gene was aligned by MAFFT (Katoh et al. 2002). The phylogenetic tree was constructed using RAxML (Stamatakis 2014) with GTR + G + I model in the CIPRES Science Gateway (Miller et al. 2010).

The plastome sequence of *L. wasingtonianum* (GenBank accession number MG590100) was 151,967 bp long, with a large single copy region of 81,393 bp, a small single copy region of 17,352 bp, and two inverted repeat regions of 26,611 bp each. Total 133 genes were identified, including 83 coding genes, 8 ribosomal RNAs, 38 transfer RNAs, and 4 pseudogenes. Among four pseudogenes, pseudo *ndhF* gene was the first to report among *Lilium* species so far.

The phylogenetic position of *L. washingtonianum* was sister to *L. superbum* but they did not form a clade with *L. philadelphicum* (Figure 1), which have belonged to section *Pseudolirion* as well as *L. washingtonianum* and *L. superbum* (Comber 1949). Interestingly, *L. philadelphicum* and *L. catesbaei* differ from other species in section *Pseudolirion* by upright flowers and immediate epigeal germination (Pelkonen and Pirttila 2012). Our results support that *L. philadelphicum* should be excluded from the section *Pseudolirion* in terms of sequence data as well as phenotypic data.

**Figure 1.**
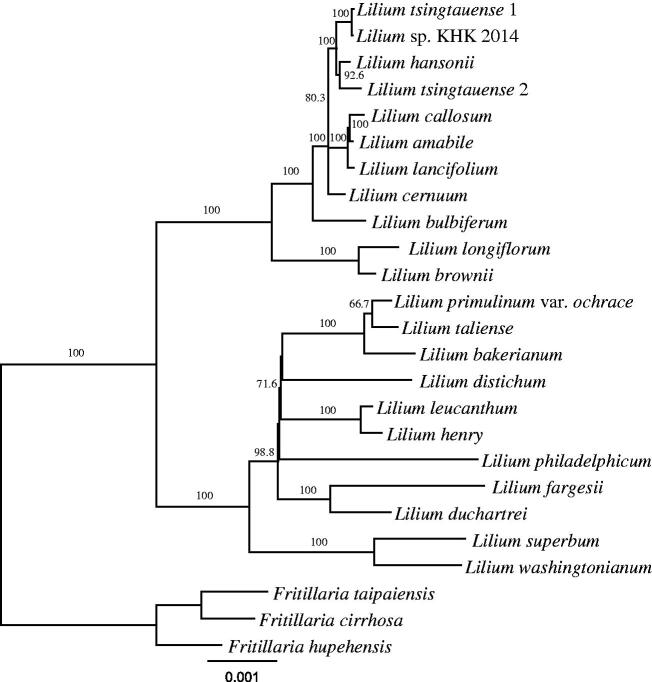
Maximum likelihood phylogenetic tree using 77 genes from 22 *Lilium* and 3 *Fritillaria* species. The *Fritillaria* species were used as outgroup. The numbers on the node and scale refer to bootstrap values and substitutions per site, respectively.
